# Supplementary material for: Head and Neck Cancer Types and Risks of Cervical–Cranial Vascular Complications within 5 Years after Radiation Therapy
Source: J Pers Med. 2022 Jun 29;12(7):1060. doi: 10.3390/jpm12071060 (PMC9317699; doi:10.3390/jpm12071060)
Supplement: Supplementary file 1 [file jpm-12-01060-s001.zip › jpm-1756393-supplementary.pdf]

Supplementary Table S1. Associated factors of significant CAS in Cox regression models

	Univariate			*Multi-variates*		
Variable	Crude HR	95% CI of HR	*p*-value	Adjusted HR	95% CI of HR	*p*-value
Nasopharyngeal carcinoma	0.10	0.02-0.43	0.002	0.17	0.05-0.57	0.004
Re-irradiation	3.32	0.58-19.09	0.18	5.95	1.25-28.29	0.03
Glycated hemoglobin	1.03	1.00-1.07	0.03	1.03	1.01-1.06	0.01
Proton beam therapy	1.30	0.39-4.38	0.67			
Coronary artery disease	11.42	0.85-153.03	0.07			
Hypertension	0.24	0.08-0.72	0.01			
Smoking	0.62	0.22-1.81	0.38			
Diabetes mellitus	0.57	0.12-2.58	0.46			
Dyslipidemia	0.42	0.10-1.81	0.25			
Radiation dose	1.00	1.00-1.00	0.34			

CAS, carotid artery stenosis; CI, confident interval; HR, hazard ratio.
